# Three-Dimensional Visualization Technology Used in Pancreatic Surgery: a Valuable Tool for Surgical Trainees

**DOI:** 10.1007/s11605-019-04214-z

**Published:** 2019-04-22

**Authors:** Chen Lin, Junyi Gao, Hua Zheng, Jun Zhao, Hua Yang, Guole Lin, Hanzhong Li, Hui Pan, Quan Liao, Yupei Zhao

**Affiliations:** 1 Department of General Surgery, Peking Union Medical College Hospital (PUMCH), Chinese Academy of Medical Sciences & Peking Union Medical College (CAMS & PUMC), Beijing, 100730 China; 2 National Virtual Simulation Laboratory Education Center of Medical Sciences, PUMCH, CAMS & PUMC, Beijing, 100730 China; 3 Eight-year Program of Clinical Medicine, PUMCH, CAMS & PUMC, Beijing, 100730 China; 4 Department of Education, PUMCH, CAMS & PUMC, Beijing, 100730 China; 5 Department of Head and Neck Surgery, PUMCH, CAMS & PUMC, Beijing, 100730 China; 6 Department of Urology Surgery, PUMCH, CAMS & PUMC, Beijing, 100730 China

**Keywords:** 3D imaging, Pancreatic cancer, Resectability evaluation, Surgical training

## Abstract

**Purpose:**

Three-dimensional (3D) visualization technology has been increasingly applied in patient-specific surgeries, but its value in residency training has not been determined. This study aimed to explore the value of 3D visualized pancreatic model in tumor evaluation and surgery planning for surgical trainees.

**Methods:**

Eighty-eight surgical residents were randomized into two groups (computed tomography (CT) group and 3D group). Both groups began with a training on evaluating the resectability of pancreatic tumor, which was based on the NCCN clinical practice guidelines and practiced on a sample case. Then, they respectively learned the sample case either on 3D reconstruction visualization tables or CT images. Finally, both groups completed a same test consisting of two pancreatic cases with CT images as well as questionnaires.

**Results:**

No differences were found in scores of the anatomy and diagnosis part, while mean scores for questions, associated with tumor staging and surgery planning, were consistently and significantly higher in the 3D group. In addition, participants in 3D group agreed that 3D technology was more beneficial in understanding and making pancreatic surgery planning.

**Conclusion:**

The 3D visualization table may be a potential supplemental learning tool in building anatomy-image-surgery knowledge system and thus making surgery planning for surgical trainees.

## Introduction

Pancreatic cancer, one of the most lethal cancers, is characterized by aggressive invasion and early metastasis.^1^ Complete resection offers the only chance of relatively long-term survival. Success of pancreatectomy depends on detailed knowledge of tumor anatomy as well as its relationship with the adjacent tissues. Errors in surgery planning may raise risk of surgical failure and recurrence. Thus, precise evaluation of tumor resectability is crucial for pancreatic surgery planning.

With the rapid development of digital technologies, 3D visualization systems have shown increasing potential value in surgery and medical education. A series of studies indicated that 3D anatomy models have been widely used in surgical teaching, patient education, and preoperative planning.^2–6^ However, a 3D-based standardized surgical training for residents is relatively lacking. Depending on this, in this study, we developed a 3D training of pancreatic cancer via 3D real-time reconstruction multi-touch visualization table to evaluate its value among surgical trainees in the anatomy-image-surgery knowledge system building and surgery planning.

## Materials and Methods

### Generating 3D Simulation Models with 3D Technology

In this study, 3D reconstructions were created by a 3D multi-touch visualization table (MVT), which was introduced by Sectra in 2010 at the Radiological Society of North America. CT data of three pancreatic cancer cases were imported into the MVT first, and real-time 3D simulation models were consequently reconstructed. All the reconstructed models were checked and assessed by two pancreas surgeons from the pancreatic surgical center of Peking Union Medical College Hospital (PUMCH), and were used in the following training and tests.

### Training on Evaluating Pancreatic Tumor Resectability

All residents participating in this study were firstly trained on evaluating the resectability of pancreatic tumors, which was based on the NCCN clinical practice guidelines in Oncology: Pancreatic Adenocarcinoma (2015.V2). Then, a clinical case of pancreatic cancer was taken as a sample for the residents to practice tumor evaluation and surgery planning with conventional continuous axial CT images. The total time was 40 min.

### Randomized Grouping

All participants were from the Department of Surgery in PUMCH. Stratified randomization was employed with two factors. Since different postgraduate years (PGYs) or genders may have influence on the remarks, years of training (PGY1, PGY2, PGY3, and PGY4) and genders were both used as stratification factors. Zelen’s algorithm was used by an invited staff from Department of Education without any conflict of interest and signed confidentiality agreement.^7^ Besides, the invited staff did not participate in the subsequent study.

### Self-Directed Learning with 3D Models or 2D CT Images

**Fig. 1:**
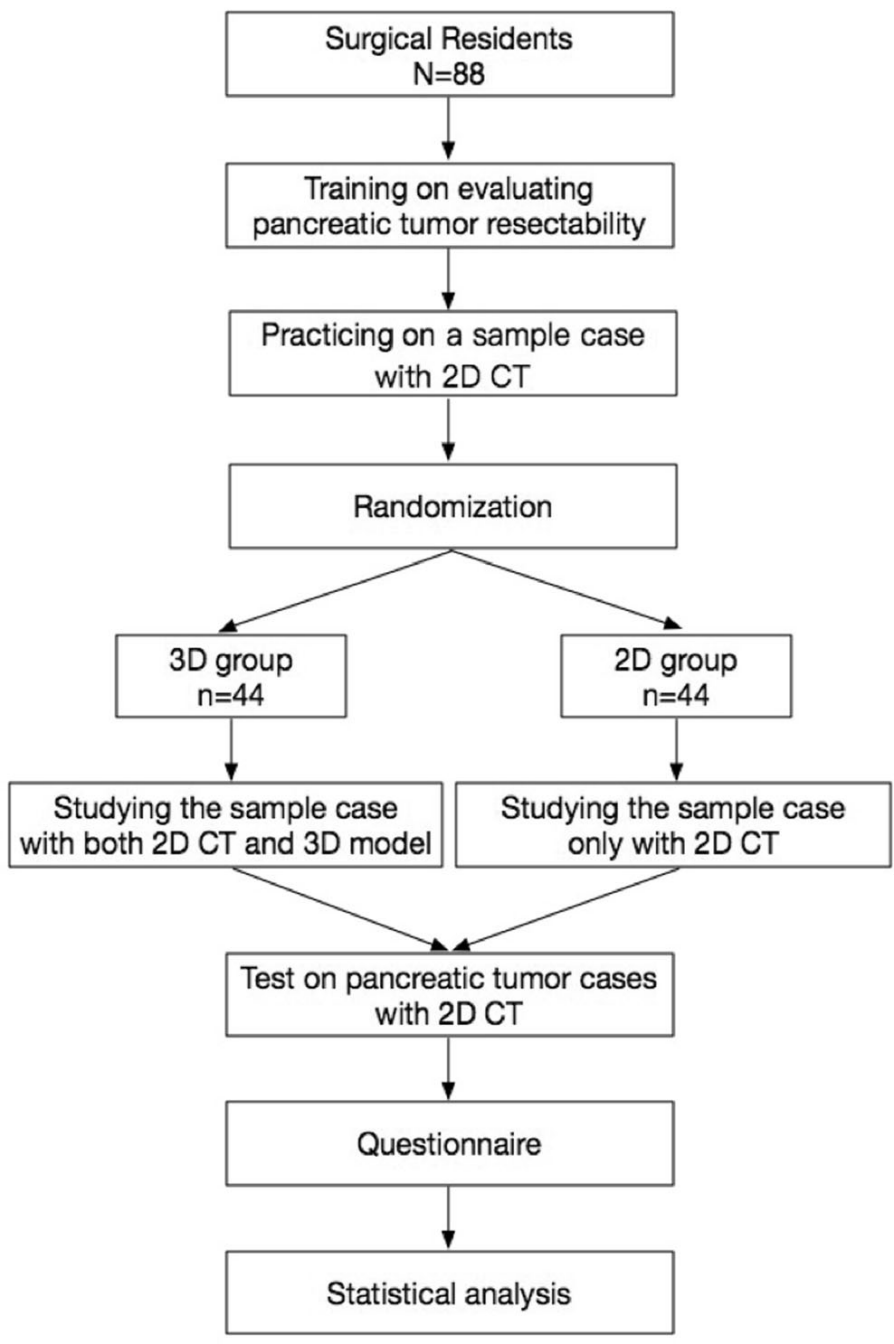
Flow chart of the study design

After the basic training, the residents in 3D group were divided into several subgroups to learn the 3D reconstruction model of the sample case on the MVTs for half an hour, and meanwhile the 2D group continued studying this sample case with the axial CT images (Fig. 1).

### Imaging Test and Questionnaire

After the theoretical learning and practice with 2D CT or 3D pancreatic models, both groups completed a same test consisting of two pancreatic cases with continuous axial CT images. Fourteen questions in aspects of anatomy, diagnosis, tumor staging, and surgery planning were developed by a group of pancreatic surgeon experts and were contained in each case (Table 1). Each question had one to four points that corresponded to detailed answers. Possible scores for each case ranged from 0 (all incorrect) to 14 (all correct). Each test paper was graded automatically by the test system.

**Table 1:** Questions for the test

Case 1: A 53-year-old woman was admitted with a 1-month history of obstructive jaundice. The level of carbohydrate antigen (CA) 19-9 was elevated up to 1200 U/mL. CT images were showed below. Please answer the following questions:			
Case 2: A 69-year-old man was admitted with a half-year history of upper abdominal pain and unintentional weight loss. CT images were showed below. Please answer the following questions:			
Questions		Options	
Anatomy and diagnosis	1. Tumor location	① Head and uncinate of pancreas ② Body of pancreas ③ Tail of pancreas	
	2. Which of the arterial phase images showed the upper bound of the tumor?	Image number:	
	3. Which of the arterial phase images showed the lower boundary of the tumor?	Image number:	
	4. Is the tumor margin clear?	① Yes	② No
	5. Is there high possibility of diagnosis of pancreatic cancer?	①Yes	② No
Tumor staging	6. Is there evidence of distant metastases in abdomen?	① Yes	② No
	7. Is there evidence of bile duct involvement?	① Yes	② No
	8. Is there evidence of lymph node enlargement?	① Yes	② No
	9. Is there evidence of tumor embolism in vein?	① Yes	② No
Surgery planning	10. Is there artery variations?	① Yes	② No
	11. Which of the following vessels might be involved?	① Abdominal aorta ② Celiac trunk ③ Splenic artery ④ Common hepatic artery ⑤ Hepatic property artery ⑥ Right hepatic artery ⑦ Left hepatic artery ⑧ Gastroduodenal artery ⑨ Superior mesenteric artery ⑩ Left renal artery ⑪ Right renal artery ⑫ Portal vein ⑬ Superior mesenteric vein ⑭ Inferior mesenteric vein ⑮ Splenic vein ⑯ Inferior vena cava ⑰ Left renal vein ⑱ Right renal vein None of above	
	12. Is there a high possibility to do the artery/vein reconstruction?	①Yes	②No
	13. Which of the following organs might be involved?	① Stomach ② Duodenum ③ Colon ④ Spleen ⑤ Small intestine ⑥ Adrenal gland ⑦ Kidney ⑧ None of above	
	14. What’s your evaluation on tumor resection?	① Resectable ② Borderline resectable ③ Unresectable	

At the end of the test, all residents completed a questionnaire, which was based on questionnaires of the System for the Evaluation of the Teaching Qualities (SETQ) and several previous studies about subjective measures toward simulation-based education.^8–10^ The questionnaire was designed to assess the residents’ attitudes toward the effects of this training on anatomy, imaging, and surgery. All the questions were rated on a 5-point Likert-type scale (Table 2).

**Table 2:** Subjective evaluation questionnaires (1-strongly disagree, 5-strongly agree) and feedback results*.* Data presented as mean ± SD

Questionnaires for surgical training			Feedback results			
			Mean scores			p*
			3D	2D	Mean difference (95% CI)	
Intention to the training	1. This is the best teaching pattern.		4.48 ± 0.55	4.32 ± 0.64	0.16 (− 0.09 to 0.41)	0.21
	2. This teaching pattern is efficient in transferring information.		4.50 ± 0.51	4.34 ± 0.64	0.16 (− 0.09 to 0.40)	0.20
	3. For 3D group: More time should be allocated to 3D models learning. For 2D group: More time should be allocated to CT learning.		4.80 ± 0.41	3.98 ± 0.79	0.82 (0.55 to 1.09)	< 0.001
	4. It is necessary to introduce this training into surgical resident program. (This question is only for 3D group)		4.75 ± 0.49	/	/	/
Anatomy	5. This training makes complex anatomy easier.		4.73 ± 0.45	4.20 ± 0.73	0.52 (0.26 to 0.78)	< 0.001
	6. This training improves understanding of the anatomic relationship between tumors and adjacent tissues.		4.48 ± 0.51	3.86 ± 0.85	0.61 (0.32 to 0.91)	< 0.001
Image reasoning	7. This training makes it easier to identify the corresponding structures in the cross-sectional CT.		4.57 ± 0.59	4.73 ± 0.54	− 0.16 (− 0.40 to 0.08)	0.19
Surgery	8. This training is beneficial for surgery planning.		4.55 ± 0.55	4.66 ± 0.57	− 0.11 (− 0.35 to 0.12)	0.34
	9. This training increased your interest in pancreatectomy.		4.70 ± 0.46	4.07 ± 0.76	0.64 (0.37 to 0.90)	< 0.001
Training modes	10. Choose your favorite learning modes:		/	/	/	/
	① Self-learning	② Group learning				
	③ Conventional lectures	④ Other modes				

*p value by unpaired *t* test

### Statistical Analysis

The distribution of continuous data was calculated, and the data were described as the mean ± standard deviation (SD). A two-sided unpaired Student’s *t* test was applied to evaluate the influence of the 3D visualized pancreatic model on the sum scores, and the level of statistical significance was set at a *p* value of < 0.05. Chi-square test was used to compare categorical variables among the cohorts. Statistical analyses were performed using SPSS (version 23.0 for Mac).

### Ethical Approval

Ethical approval was obtained from the Institutional Review Board of the Institute of Basic Medical Sciences, Chinese Academy of Medical Sciences (Project No. 009-2014). All participants completed written informed consent. Study methods were performed in accordance with approved guidelines.

## Results

### Characteristics of Residents

A total of 88 surgical residents (77 males and 11 females) participated, and there was no prior exposure to the Sectra table. Forty-four residents, consisting of 19 PGY1, 12 PGY2, 9 PGY3, and 4 PGY4 residents, were randomized into the 3D group, and 44 residents, consisting of 20 PGY1, 11 PGY2, 10 PGY3 and 3 PGY4 residents, were grouped into the CT group. In the 3D model group, 25% of the resident had previously assisted in pancreatic surgeries, and 23% in the 2D CT group had the same experience. There were no statistically significant differences in gender (*p* = 0.747), PGYs (*p* = 0.967), or previous pancreatic surgery experience (*p* = 0.803) (Table 3).

**Table 3:** Baseline characteristics of residents*.* All values presented as *n* (%). *p value by chi-square test

		3D model + CT, *n* = 44	2D CT, *n* = 44	p*
Years of residency training	1	19 (43)	20 (45)	0.967
	2	12 (27)	11 (25)	
	3	9 (21)	10 (23)	
	4	4 (9)	3 (7)	
Gender	Male	38 (86)	39 (89)	0.747
	Female	6 (14)	5 (11)	
Pancreatic surgery experience, yes		11(25)	10(23)	0.803

### Scores of the Test

Overall, the mean scores for questions, in aspects of tumor staging and surgery planning, were significantly higher in the 3D group compared with the 2D group for both cases (case 1: 3D vs. CT, ①Tumor staging: mean difference (MD) = 0.68, *p* < 0.001. ②Surgery planning: MD = 1.00, *p* < 0.001. case 2: 3D vs. CT, ①Tumor staging: MD = 0.84, *p* < 0.001. ②Surgery planning: MD = 0.93, *p* < 0.001), whereas significant differences were not observed regarding with the scores in the anatomy and diagnosis part (3D vs. CT, case 1: MD = 0.32, *p* = 0.06. case 2: MD = 0.25, *p* = 0.14.) (Table 4).

**Table 4:** Mean scores for each test question. Data presented as mean ± SD. *p value by unpaired *t* test

Questions	Test scenario	3D group *n* = 44	2D group *n* = 44	Mean difference (95% CI)	p*
Anatomy and diagnosis	Case 1	3.91 ± 0.80	3.59 ± 0.79	0.32 (− 0.02 to 0.65)	0.06
	Case 2	3.55 ± 0.85	3.30 ± 0.73	0.25 (− 0.09 to 0.59)	0.14
Tumor staging	Case 1	3.09 ± 0.71	2.41 ± 0.76	0.68 (0.37 to 0.99)	< 0.001
	Case 2	3.27 ± 0.73	2.43 ± 0.62	0.84 (0.55 to 1.13)	< 0.001
Surgery planning	Case 1	3.36 ± 0.72	2.36 ± 0.89	1.00 (0.66 to 1.34)	< 0.001
	Case 2	3.39 ± 0.69	2.45 ± 0.93	0.93 (0.59 to 1.28)	< 0.001
Sum scores	Case 1	10.36 ± 1.38	8.36 ± 1.42	2.00 (1.41 to 2.59)	< 0.001
	Case 2	10.20 ± 1.32	8.18 ± 1.24	2.02 (1.48 to 2.57)	< 0.001

### Answers to the Questionnaire

The feedback for the questions (Table 2) related to anatomy indicated that the 3D reconstructed models had great value in improving understanding of complex structures (3D vs. 2D. question 5: MD = 0.52, *p* < 0.001; question 6: MD = 0.61, *p* < 0.001). However, both trainings were regarded as being helpful in imaging reasoning and surgery planning (question 7: MD = − 0.16, *p* = 0.19; question 8: MD = − 0.11, *p* = 0.34). When asked about their personal attitudes toward these trainings, residents from both groups considered that the learning pattern they just underwent was the best and was efficient in information transfer (question 1: MD = 0.16, *p* = 0.21; question 2: MD = 0.16, *p* = 0.20). In addition, residents from the 3D group agreed to allocate more time to the 3D models learning part, while the 2D group thought that time for CT interpretation was enough (question 3: MD = 0.82, *p* < 0.001). Feedback for question about surgery indicated that training containing 3D models significantly increased residents’ interest in surgery, and residents trained with 3D models highly agreed on introduction of this tool into the surgical resident program (question 4: mean score = 4.75 ± 0.49; question 9: MD = 0.64, *p* < 0.001.).

## Discussion

Surgery is currently the only potential curative method for pancreatic cancer. Because of the anatomical complexities and variations of vessels, the success of the treatment depends on a precise evaluation of the tumor resectability and surgery planning. With the rapid development of digital technologies, 3D visualization system has shown increasing potential value in patient-specific surgeries for its advantages in improving intuition and simplifying complex structures. Application of 3D liver models has revealed their value in precise hepatectomy, in which 3D liver models, based on CT or MR images from clinical patients, facilitated the preoperative planning, intraoperative identification, and resection of liver and vessels.^11, 12^ Another study, exploring the effects of 3D reconstruction of peripancreatic vascular system on pancreatic surgeries, found that this novel application could apparently reduce surgical trauma and decrease operative time.^13^ Although the 3D technology has achieved initial success in clinical application, it is still not systematically introduced into the standardized residency training. Thus, we designed this study to explore the value of 3D visualized pancreatic model in tumor evaluation and surgery planning for surgical trainees.

**Fig. 2:**
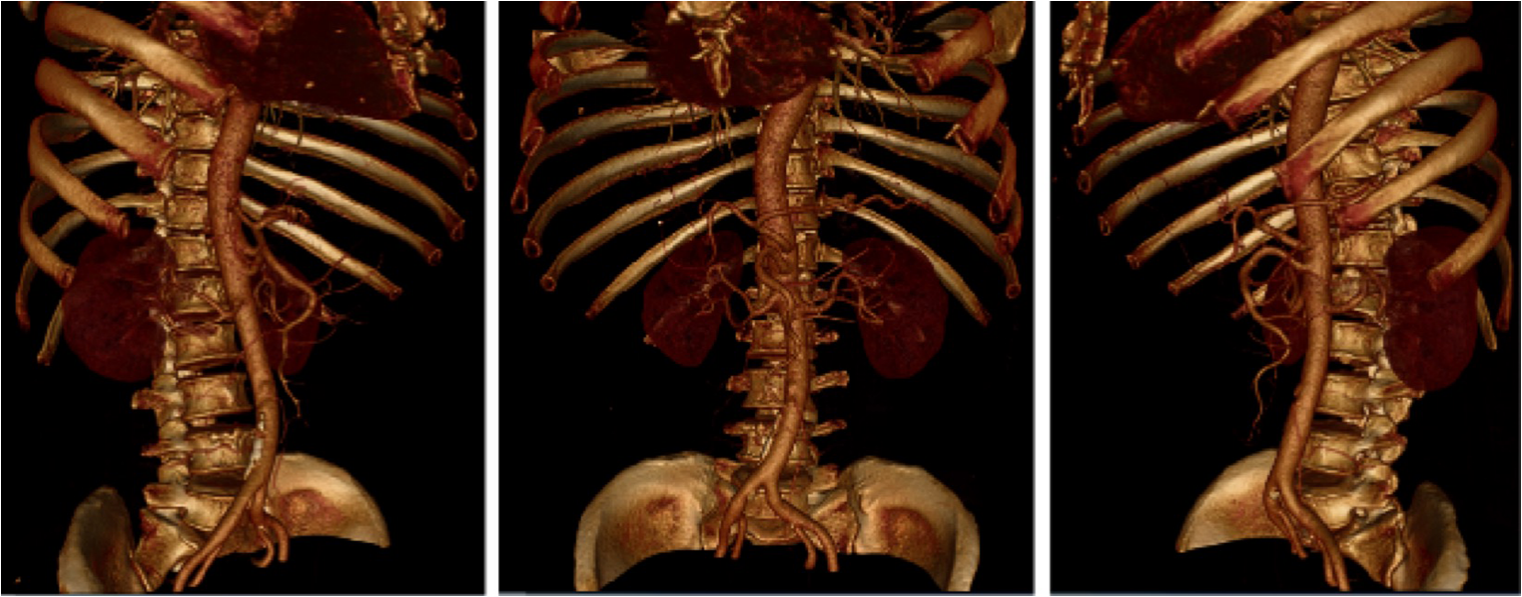
Peripancreatic arteries in the artery preset

**Fig. 3:**
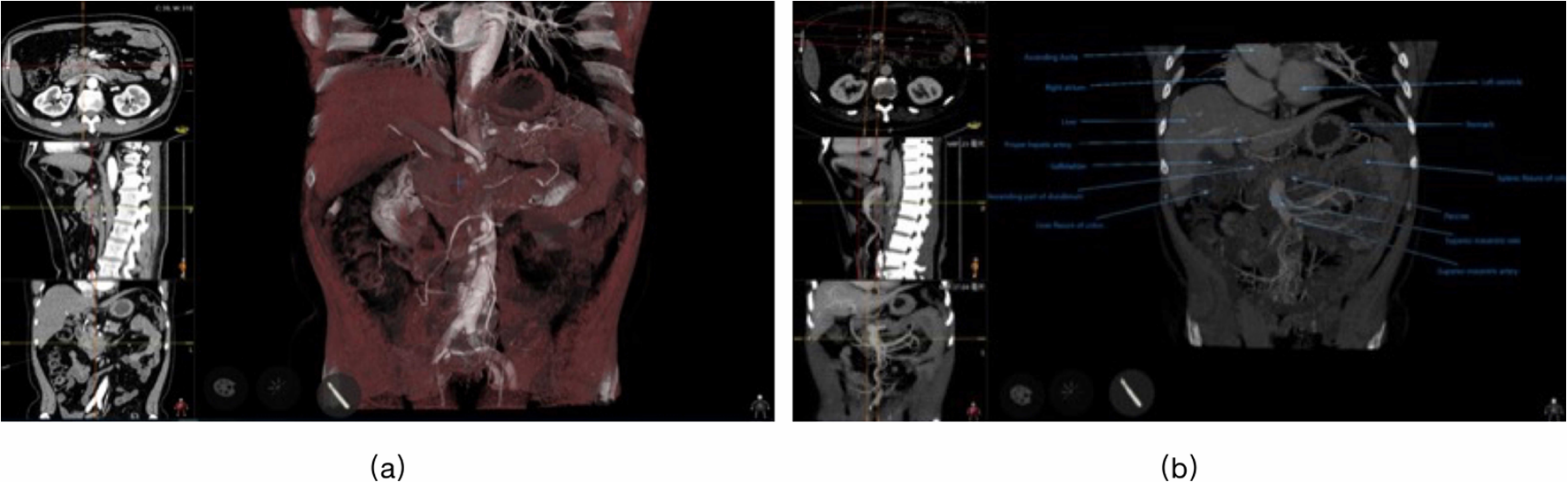
Tumor and adjacent arteries in the **a** artery-organ preset and **b** vein-organ preset. Location of the pancreatic tumor was marked on the coronal, axial, and sagittal CT images with cross cursors, and the blue marker indicates its corresponding location in the 3D reconstructed model

During the training, residents from the 3D group interacted with pancreatic tumor models on the MVTs in the following order: (1) To identify peripancreatic arteries in artery preset. Trainees traced the major peripancreatic arteries in the 360 degree-view reconstructed model and compared the corresponding structures in the 2D CT images on the coronal, axial, and sagittal planes via the crosshair function (Fig. 2). (2) To clarify the spatial relationship between the tumor and adjacent arteries in the artery-organ preset via the crosshair function again. Based on identification of major arteries and tumors previously, trainees inferred the precise location of tumors and their spatial relationship with adjacent arteries in this model. In addition, they simulated surgical procedures by slice cutting in the coronal direction (Fig. 3a). (3) To understand the spatial relationship between the tumor and adjacent arteries and veins in the vein-organ preset (Fig. 3b). It was worth mentioning that the MVT was further explored by some residents, and an air preset was developed to study the spatial relationship among other abdominal vessels and stomach and colon, which reflected its potential value in allowing trainees to be innovative and creative.

In the design of the test, to evaluate surgical trainees’ anatomy-image-surgery knowledge system, the questions were mainly classified into three groups including “anatomy and diagnosis,” “tumor staging,” and “surgery planning” for a better further analysis of the corresponding drill. Both groups used 2D CT images during the test, considering that according to the NCCN guidelines for pancreatic adenocarcinoma, decisions about diagnostic management and resectability rely on the 2D image, that appropriate high-quality imaging studies including pancreatic CT (preferred) or MRI with contrast. 3D image could be as additional reference. Thus, although 3D group were trained with 2D and 3D images, the test was still designed with 2D images of pancreatic CT. Moreover, the learning pattern of CT images could be summarized as the process of “2D→3D→2D,” in which 3D structures primarily formed through cross-sectional 2D CT images in the trainees’ mind, and the following CT interpretation was based on the 3D structures that were previously built. Depending on this, 3D training was given to the surgical residents to improve the spatial learning and accurate understanding of the anatomic relationship between tumors and adjacent tissues, via the real-time corresponding learning between 2D and 3D structures. In addition, designing the tests without 3D models might better assess the effects of 3D training on the reconstruction process of the surgical trainees.

Results of the present study showed that residents in the 2D group had lower scores for both two test cases, which might be an indirect evidence that the visual-spatial ability of surgical residents may not be adequate to interpret 2D to 3D images accurately. Moreover, residents after training with 3D real-time reconstructed models had a better performance in more complex clinical questions, such as those related to tumor staging and surgery planning, which may demonstrate the advantages of 3D virtual models in simplifying complex structures and improving understanding of spatial relationship between tumors and adjacent structures.

In the feedback of questionnaires, from questions 1 and 2, it was interesting to observe that trainees in both 2D group and 3D group considered their training pattern which they have had as the best teaching pattern and efficient information transfer method. A possible explanation might be that both trainings indeed improved participants’ knowledge in anatomy, imaging reasoning, and surgery planning, which could be demonstrated by the high scores of corresponding test questions. However, the significant differences between test scores of tumor staging and surgery planning might provide insight in the effect of 3D models on improving residents’ ability in tumor evaluation and treatment decisions. Another reason for this was that high acceptability toward the conventional CT training among the trainees in the 2D group might be partly related to their lack of knowledge on the effect of 3D virtual technology combined with pancreatic surgery planning training.

The Sectra table is an ergonomic, multi-touch display workstation that lets users simply touch the screen and interact with the 3D models intuitively. During the 3D models training part, residents interacted with 3D models in small groups, and everyone immersed himself or herself in the operation and discussion from beginning to the end. This result was also confirmed by the feedback of the questionnaires, as residents from 3D group agreed to allocate more time to the self-learning part, while feedback from the 2D group was negative. This difference indirectly indicated that the virtual table might add more interest to the process of building the anatomy-image-surgery knowledge system. In addition, feedback of question about training modes showed a preference to the group learning and case-based learning patterns. These suggestions inspired us to develop a more interesting and efficient training mode combining real clinical cases with group-learning patterns in the future.

Regarding the cost, there was limited study about the comparison between traditional and 3D modalities for training purpose. However, in clinical applications, Sean S. Li et al. demonstrated a reduction in the total cost of cases of head and neck reconstruction using computer-aided surgical simulation (CASS) compared with traditional option.^14^ In our study, the software and equipment costs of Sectra table were approximately $250,000. Considering the time spent from the attendings, a series of cases with key vessels for surgery marked on the Sectra table were designed, with an average of 1–2 h per case, and surgical residents can self-learn in mini groups, without the supervision of the attending physician. The attending physician can also introduce real-life cases into the 3D imaging system for direct face-to-face training without the need for more time to prepare.

Our study has several limitations. First, this is a single-center study in a leading teaching hospital of the country and is biased by center selection. In the future, multi-center study can help improve our current knowledge of the potential value of introduction of 3D technology into pancreatic surgery planning training during different levels of centers and extend its application in other subspecialties. Besides, a systematic training program designed with more training time and including more hepato-pancreato-biliary tumor cases can give us deeper insight into the impact of 3D technology in surgical oncology training, particularly in tumor evaluation and surgery planning. Also, given that there is currently no systematic 3D surgical resident training in our institution, complete data was not collected on this variable, previous 3D training time, which could be considered in the future multi-center study.

## Conclusion

This randomized study revealed that the 3D real-time visualization table may have the potential to be a valuable supplemental learning tool in building anatomy-image-surgery knowledge system and thus making surgery planning for surgeon trainees, as it provided a better 3D understanding of the tumor and its surroundings and demonstrated advantages for interacting with cross sectional images. Therefore, it is recommended that this 3D visualization technology, especially with real clinical cases, be systematically introduced into surgical residency for pancreatic surgery training.
